# What Is the Most Suitable Agent Combined With Apatinib for Transarterial Chemoembolization Treatment in Advanced Hepatocellular Carcinoma Patients? A Systematic Review and Network Meta-analysis

**DOI:** 10.3389/fonc.2022.887332

**Published:** 2022-05-25

**Authors:** Fuhai Hui, Chang Xu, Xiangbo Xu, Jiangxia Chen, Hefeng Geng, Chao Yang, Yingshi Zhang

**Affiliations:** ^1^Department of Clinical Pharmacy, Shenyang Pharmaceutical University, Shenyang, China; ^2^Department of Ethnic Culture and Vocational Education, Liaoning National Normal College, Shenyang, China

**Keywords:** transarterial chemoembolization (TACE), apatinib, advanced hepatocellular carcinoma, systematic review, network meta-analysis

## Abstract

**Purpose:**

Combined therapy with transarterial chemoembolization (TACE) and apatinib is superior in therapeutic effect compared with TACE alone in patients with hepatocellular carcinoma (HCC). To determine the most suitable agent combined with apatinib for TACE treatment, we did a systematic review and network meta-analysis.

**Methods:**

Four electronic databases were searched from inception until November 2021. Randomized controlled trials (RCTs) and retrospective studies that combined therapy of TACE and apatinib (TACE+A) compared with TACE alone were included. We performed random-effect pairwise and network meta-analyses to summarize the outcomes about efficacy and safety.

**Results:**

Forty-five original studies including 3,876 patients were included. In terms of efficacy, we evaluated treatment response, 6 months overall survival (OS), 1 year OS, 6 months progression-free survival (PFS), 1 year PFS, alphafetoprotein (AFP), matrix metalloproteinase 9 (MMP9), and vascular endothelial growth factor (VEGF). Significant differences always appear in TACE agent subgroups of adriamycin, platinum, and fluorouracil from both pairwise and network meta-analysis, while significant differences could also be found in apatinib dosage of 500 and >500 mg/day subgroups and in both RCT and retrospective study subgroups. From second time network analysis, compared with TACE alone, subgroups with TACE agents of oxaliplatin, cisplatin, pirarubicin, epirubicin, and 5-fluorouracil ranked front. In addition, the safety of adriamycin, platinum, and fluorouracil subgroups is acceptable.

**Conclusions:**

In conclusion, the most suitable agents in TACE combined with apatinib were adriamycin+platinum ± fluorouracil combination therapy.

**Systematic Review Registration:**

The study was registered with https://www.crd.york.ac.uk/PROSPERO/display_record.php?RecordID=311650, PROSPERO, CRD4202022311650

## Introduction

Liver cancer is a highly malignant tumor, and its morbidity and mortality are increasing year by year, ranking sixth and fourth in the world, respectively. Liver cancer includes two histological types, namely, hepatocellular carcinoma (HCC) and intrahepatic cholangiocarcinoma (ICC), of which HCC accounts for as high as 85%–90% (1). HCC has a high degree of deterioration, insidious onset in the early stage, and rapid development in the middle stage. Most of the patients are in the middle and late stage when they are clinically diagnosed (2). At present, local interventional therapy has been recognized as one of the preferred treatment methods of patients with intermediate-stage HCC.

Multiple treatments, such as transarterial chemoembolization (TACE), transarterial radioembolization (TARE), percutaneous ethanol injection (PEI), and radiofrequency ablation (RFA), have been used as downstaging treatments (3). In recent years, transarterial chemoembolization (TACE) has been widely used, which is the most representative local treatment method in minimally invasive treatment of HCC. TACE is the first-line treatment for patients with intermediate-stage HCC, including those with large or multinodular HCC, well-preserved liver function, and no cancer-related symptoms or evidence of vascular invasion or extrahepatic spread (4). TACE has the advantages of convenient operation, less trauma, and accurate curative effect.

There are two first-line targeted treatment options for advanced HCC, namely, sorafenib and lenvatinib, and three second-line targeted treatment options, namely, regorafenib, cabozantinib, and ramucirumab (5–9). However, the application of molecular targeted agent therapy after local treatment often leads to treatment failure due to the easy recurrence of tumors. All of the above are the main factors for the low 5-year survival rate (only 20%) of patients with advanced HCC. Therefore, confirming new and more effective molecular targeted agent to prevent HCC metastasis and recurrence is of great clinical value (10, 11). Thus, another molecular targeted agent apatinib is widely used in HCC as an alternative. Apatinib inhibits tumor angiogenesis by targeting the vascular endothelial growth factor-2 (VEGFR-2). Apatinib was approved for use in China in 2014 for the treatment of advanced gastric adenocarcinoma. Apatinib was approved for increased use in China for the treatment of patients with advanced HCC who had previously failed or were intolerant to at least one first-line systemic therapy in December 2020. TACE in combination with Apatinib was superior to TACE alone in the long-term treatment of advanced HCC (12, 13), while apatinib could be used as second-line treatment in advanced HCC therapy. However, the most suitable agent combined with apatinib in TACE treatment needs to be identified. No previous systematic review has provided a comprehensive overview with pairwise and network meta-analysis evaluating which type of agent in TACE is the most suitable in combination with apatinib.

### Materials And Methods

This systematic review and network meta-analysis followed the guidelines of the Preferred Reporting Items for Systematic Reviews and Meta-analyses (PRISMA) checklist (14), and the protocol was registered with the international prospective register of systematic reviews (PROSPERO, https://www.crd.york.ac.uk/PROSPERO/display_record.php?RecordID=311650) with registration number CRD4202022311650 (15).

### Search Strategy and Eligibility Criteria

We systematically searched electronic databases of PubMed, Embase, the Cochrane Library, and the China National Knowledge Infrastructure (CNKI) from inception until January 2022, using the following search terms of apatinib, transarterial chemoembolization, hepatocellular carcinoma, and their MeSH terms without language restrictions. Two independent researchers (HH and XC) screened possible inclusion of publications and extracted data, and any disagreement and controversy were resolved by consensus with the third experienced researcher (ZS). Randomized controlled trials (RCTs) and retrospective studies that met the inclusion criteria of TACE plus apatinib (TACE+A) treatment versus TACE treatment alone in patients with HCC were incorporated. Studies with no survival data were also excluded. Moreover, the reference lists of relevant systematic reviews were also identified for potential eligible studies.

### Data Collection and Risk of Bias

For each eligible study, the above pairs of studies extracted data independently using a standardized table. Baseline characteristics such as first author, publication year, trial type, sample size, age, hepatitis B virus (HBV) infection, tumor–nodes–metastasis (TNM) stage, tumor size, Barcelona clinic liver cancer (BCLC) stage, Child–Pugh classification, Eastern Cooperative Oncology Group (ECOG) score, cycles of TACE, apatinib dosage, and TACE agent were extracted. Besides, efficacy evaluations included treatment response (TR), 6 months overall survival (6M-OS), 1 year OS (1Y-OS), 6 months progression-free survival (6M-PFS), 1 year PFS (1Y-PFS), alphafetoprotein (AFP), matrix metalloproteinase 9 (MMP9) and VEGF. Patients had to have a measurable disease as defined by the Response Evaluation Criteria in Solid Tumors (version 1.1; RECIST v1.1), which defined the percentage of patients achieving best overall response of either a complete response (CR), partial response (PR), or stable disease (SD) (16). Safety evaluations included hypertension, hand–foot syndrome, fatigue, fever, nausea–vomiting, and diarrhea. Moreover, subgroup analysis was conducted according to the classification of TACE agent (adriamycin+platinum, adriamycin+platinum+fluorouracin, platinum+fluorouracin, platinum+raltitrexed, adriamycin+fluorouracin, adriamycin, and fluorouracil), apatinib dosage (500, >500, and <500 mg/day), and trial type (RCT and retrospective study).

For eligible RCT, the Cochrane tool for assessing risk of bias (RoB 2.0) (17) was used to evaluate RCTs as follows: all low-risk domains were considered as low-risk research, one high-risk domain was considered as high-risk research, and another was considered as unknown risk research. The quality of eligible retrospective study was assessed using the Newcastle–Ottawa Scale (NOS) score (18), and the score >4 is acceptable. We also used the grading system of Recommendations Assessment, Development and Evaluation (GRADE) scales (19) to evaluate the quality of the outcomes from pairwise meta-analysis.

### Data Synthesis and Analysis

For each direct comparison for each outcome, we performed a random-effects pairwise meta-analysis to avoid inconsistencies caused by different studies. The heterogeneity among studies were assessed by *p*-value and *I*^2^ statistics, and *p*-values <0.05 or *I*^2^ >50% indicated heterogeneity in the outcome (20). For all dichotomous outcomes, the odds ratios (ORs) and corresponding 95% credible intervals (95%CIs) were used to confirm the significance of meta-analysis results, and for continuous outcomes, the standardized mean differences (SMDs) and their 95%CI were applied. Moreover, the *p*-value from meta-regression was used to determine whether the factor was the source of heterogeneity, where a *p*-value <0.05 means yes (21). We performed Begg’s and Egger’s test to assess the publication bias for available comparisons, a *p*-value <0.05 means the existence of publication bias.

For network comparison of each outcome, we performed a random-effects frequencies network meta-analyses for more accuracy of data (22), and ORs and their 95%CI were applied. Inconsistency between indirect sources of evidence was statistically assessed using a global (design-by-treatment inconsistency model) and a local method (back calculation) (23, 24), and mean rank and surface under the cumulative ranking curve (SUCRA) values were produced from network meta-analysis estimates with a consistent model, which was used to rank every TACE agent. SUCRA score ranged from 0% to 100%, a higher SUCRA score indicating that there is a high possibility of becoming the most suitable TACE agent. We produced comparison-adjusted funnel plots to explore publication bias for network meta-analysis outcomes. All the aforementioned analyses were performed using StataSE version 15.1.

## Results

### Description of Included Studies

**Figure 1:**
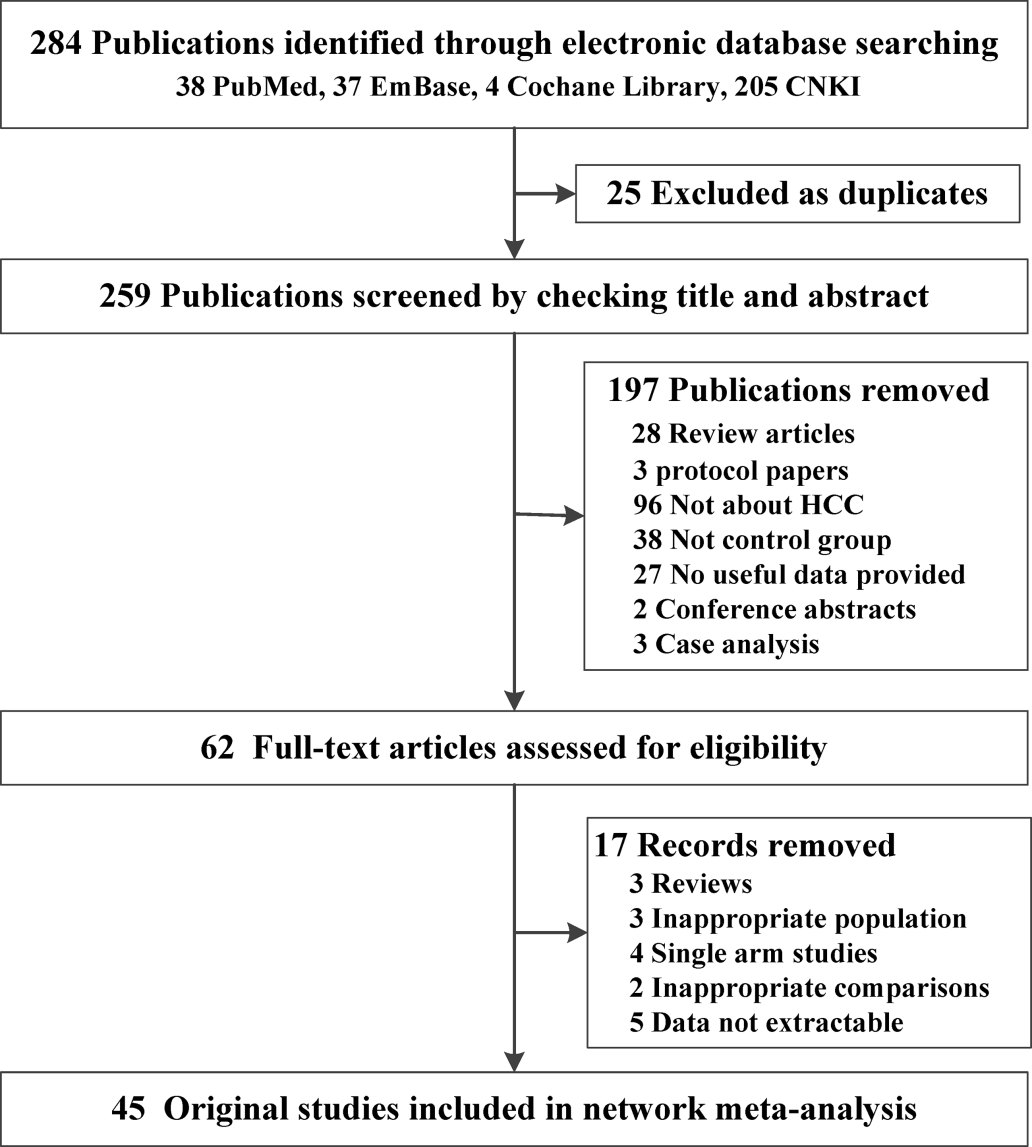
Flow chart of studies included in the review of TACE + apatinib versus TACE studies. HCC, hepatocellular carcinoma.

**Figure 1** shows the details of the original study selection process. The electronic search yielded 259 unique publications. After screening titles, abstracts, and full-text articles, 45 original studies including 3,876 patients with HCC were identified (25–69). Seventeen of the 45 studies were RCTs and the remaining 28 were retrospective studies. Moreover, a combination of three agents was used as TACE therapy in 10 studies, and nearly half of the included studies (n=21) make use of two kinds of agent in TACE therapy; 10 of the included studies only applied one kind of agent, and the other three studies did not mention the agent in TACE. In addition, most of our included studies used apatinib at a dose of 500 mg/day. For baseline indicator characteristic meta-analysis, the items of gender (M/F), age, HBV infection, tumor size (<5/≥5), BCLC stage (B/C), Child–Pugh classification (A/B–C), ECOG score (0–1/2), and cycles of TACE were all balanced between TACE+A versus TACE group (**Table 1**; **Supplementary Table 1**). In terms of inconsistency detection, the inconsistency among the included studies was acceptable (**Supplementary Figure 2**). The quality assessments of both RCT and retrospective studies were all acceptable for meta-analysis (**Supplementary Figure S3****;** **Supplementary Table S2**).

**Table 1:** Baseline characteristic of included TACE+apatinib versus TACE alone studies.

TACE agent classify	TACE agent	Apatinib dosage	Trial type	Sample size (I/C)	Reference
**Adriamycin+** **platinum+fluorouracil**	EPI+DDP+5-FU	500 mg, 2/day	Retrospective study	29/29	(26)
		750 mg, 1/day	RCT	50/50	(27)
		750 mg, 1/day	RCT	50/50	(33)
	THP+L-OHP+5-FU	750 mg, 1/day	Retrospective study	35/33	(30)
		750 mg, 1/day	RCT	40/40	(31)
		500 mg, 1/day	RCT	35/35	(53)
		500 mg, 1/day	Retrospective study	28/31	(54)
	EPI+L-OHP+5-FU	750 mg, 1/day	RCT	52/52	(34)
		500 mg, 1/day	Retrospective study	30/30	(58)
	EPI+lobaplatin+FT-207	500 mg, 1/day	Retrospective study	30/30	(44)
**Adriamycin + platinum**	EPI+lobaplatin	500 mg, 1/day	Retrospective study	86/115	(25)
		500 mg, 1/day	RCT	50/50	(63)
	EPI+L-OHP	500 mg, 1/day	RCT	50/50	(32)
		500 mg, 1/day	Retrospective study	71/81	(37)
		500 mg, 1/day	Retrospective study	28/51	(52)
		500 mg, 1/day	RCT	44/44	(68)
		850 mg, 1/day	RCT	38/38	(66)
		850 mg, 1/day	RCT	22/22	(67)
		500 mg, 1/day	Retrospective study	20/22	(69)
	EPI+L-OHP/DDP	250 mg, 2/day	RCT	45/45	(49)
	THP+Lobaplatin	500 mg, 1/day	RCT	36/36	(35)
		500 mg, 1/day	Retrospective study	20/20	(38)
		500 mg, 1/day	Retrospective study	36/28	(40)
		500 mg, 1/day	Retrospective study	29/29	(51)
	ADM+DDP	500 mg, 1/day	Retrospective study	25/25	(56)
	EPI+CBP	500 mg, 1/day	Retrospective study	85/103	(47)
**Platinum+fluorouracil**	L-OHP+5-FU	750 mg, 1/day	RCT	50/50	(28)
		500 mg, 1/day	Retrospective study	31/30	(43)
**Adriamycin+fluorouracil**	ADM+5-FU	850 mg, 1/day	RCT	20/20	(68)
**Platinum+raltitrexed**	Lobaplatin+raltitrexed	500 mg, 1/day	Retrospective study	30/30	(41)
				42/56	(45)
		300 mg, 1/day	Retrospective study	30/30	(41)
**Adriamycin**	ADM	500 mg, 1/day	Retrospective study	83/82	(29)
				90/90	(36)
				36/28	(39)
		500 mg, 1/day	RCT	23/23	(58)
	EPI	500 mg, 1/day	Retrospective study	24/24	(42)
		250 mg, 2/day	Retrospective study	108/102	(50)
		500 mg, 1/day	RCT	42/41	(55)
		250 mg, 1/day	RCT	38/52	(56)
		500 mg, 1/day	Retrospective study	28/28	(65)
	FT-207	500 mg, 1/day	Retrospective study	20/25	(57)
**Fluorouracil**	5-FU	500 mg, 1/day	Retrospective study	30/30	(66)
**No mention**		500 mg, 1/day	Retrospective study	25/22	(59)
		500 mg, 1/day	Retrospective study	25/25	(61)
		500 mg, 1/day	Retrospective study	27/53	(62)
**Baseline indicator**		**OR/SMD(95%CI)**	**Heterogeneity**	**Balance or not**	
Gender (M/F)		0.960 (0.822, 1.122)	0.469, 0%	Yes	
Age^#^		−0.069 (−0.139, 0.001)	0.572, 0%	Yes	
HBV infection		1.082 (0.814, 1.439)	0.913, 0%	Yes	
Tumor size (<5/≥5)		0.951 (0.701, 1.290)	0.604, 0%	Yes	
BCLC stage (B/C)		1.064 (0.841, 1.348)	0.705, 0%	Yes	
Child–Pugh classification (A/B-C)		1.010 (0.839, 1.216)	0.999, 0%	Yes	
ECOG score (0–1/2)		0.841 (0.609, 1.160)	0.959, 0%	Yes	
Cycles of TACE^#^		-0.146 (-0.495, 0.203)	0.000, 88.3%	Yes	

5-FU, 5-fluorouracil; ADM, doxorubicin; DDP, cisplatin; EPI, epirubicin; FT207, tegafur; L-OHP, oxaliplatin; RCT, randomized controlled trial; TACE, transarterial chemoembolization; THP, pirarubicin. ^#^SMD.

### Pairwise Meta-Analysis Outcomes of TACE + Apatinib Versus TACE Therapy in Efficacy

We considered the efficacy outcomes such as TR, 6M-OS, 1Y-OS, 6M-PFS, 1Y-PFS, AFP, MMP9, and VEGF, and these above outcomes were subgroup analyzed by TACE agent classification, apatinib dosage, and trial type. Thirty-eight of the 45 studies evaluated the outcome of TR. When TR was analyzed by TACE agent classification, significant differences could be found in overall (2.005, 1.567–2.564) and in adriamycin+platinum (2.155, 1.289–3.604), adriamycin+platinum+fluorouracin (2.118, 1.427–3.142), platinum+fluorouracin (2.098, 1.082–4.069), and adriamycin (2.089, 1.154–3.781) subgroups, with low to substantial heterogeneity among the included studies (*I*^2 =^ 0%–68.8%). When TR outcome was grouped by apatinib dosage, significant outcomes could be found in 500 mg/day (2.047, 1.488–2.815) and >500 mg/day (1.893, 1.359–2.638) subgroups with uncertain heterogeneity. Moreover, significant difference could be found in both RCT (2.414, 1.866–3.122) and retrospective study (1.759, 1.214–2.549). Meta-regression did not reveal the source of heterogeneity with *p-*value >0.05. For the overall outcomes and all subgroups, no publication bias could be found from Begg’s and Egger’s test, with very low to moderate grades (**Table 2**).

**Table 2:** Efficacy of TACE+apatinib versus TACE in treatment response, overall survival, progression-free survival, AFP, MMP9, and VEGF, which were analyzed by TACE agent classification, apatinib dosage, and trial type.

Outcomes	Subgroups	Included studies	OR(95%CI)	*p*, *I*^2^	*p* from meta-regression	*p* from publications bias	Grade
**Treatment response (TR)**	**Overall**	38	2.005 (1.567, 2.564)*	0.000, 55.6%^#^		0.706, 0.466	Low
**TACE agent classify**							
	Adriamycin+platinum	12	2.155 (1.289, 3.604)*	0.000, 68.8%^#^	0.429	0.411, 0.844	Low
	Adriamycin+platinum+fluorouracin	8	2.118 (1.427, 3.142)*	0.277, 19.3%		0.063, 0.504	Moderate
	Platinum+fluorouracin	2	2.098 (1.082, 4.069)*	0.328, 0.0%		–	Low
	Platinum+raltitrexed	3	1.807 (1.037, 3.150)	0.609, 0.0%		1.000, 0.155	Low
	Adriamycin+fluorouracin	1	3.000 (0.507, 17.740)	–		–	Very low
	Adriamycin	8	2.089 (1.154, 3.781)*	0.003, 67.0%^#^		0.108, 0.593	Low
	Fluorouracil	1	1.784 (0.616, 5.169)	–		–	Very low
	No mention	3	0.875 (0.131, 5.825)	0.001, 84.9%^#^		0.296, 0.192	Very low
**Apatinib dosage**							
	500 mg/day	28	2.047 (1.488, 2.815)*	0.000, 64.3%^#^	0.640	0.407, 0.429	Low
	>500 mg/day	9	1.893 (1.359, 2.638)*	0.556, 0.0%		0.754, 0.671	Moderate
	<500 mg/day	1	1.321 (0.469, 3.721)	–		–	Very low
**Trial type**							
	RCT	15	2.414 (1.866, 3.122)*	0.620, 0.0%	0.277	0.656, 0.811	High
	Retrospective study	23	1.759 (1.214, 2.549)*	0.000, 68.1%^#^		0.616, 0.470	Low
**Overall survival(OS)**							
**6M-OS**	**Overall**	27	2.311 (1.744, 3.062)*	0.228, 16.2%		0.466,0.136	Moderate
**TACE agent classify**							
	Adriamycin+platinum	9	2.219 (1.376, 3.579)*	0.110, 38.7%	0.505	0.251,0.139	Moderate
	Adriamycin+platinum+fluorouracin	7	2.666 (1.565, 4.540)*	0.456, 0.0%		0.230, 0.418	Moderate
	Platinum+raltitrexed	3	11.748 (2.154, 64.078)*	0.980, 0.0%		0.602, –	Moderate
	Adriamycin+fluoroura	1	2.786 (0.773,10.043)	–		–	Very low
	Adriamycin	4	1.492 (0.584, 3.812)	0.111, 50.1%^#^		0.308, 0.077	Low
	Fluorouracil	1	2.071 (0.178, 24.148)	–		–	Very low
	No mention	2	3.163 (1.124, 8.902)*	0.496, 0.0%		–	Low
**Apatinib dosage**							
	500 mg/day	18	2.305 (1.604, 3.311)*	0.157, 25.4%	0.958	0.344, 0.138	Moderate
	>500 mg/day	7	2.676 (1.587, 4.511)*	0.456, 0.0%		0.099, 0.412	Moderate
	<500 mg/day	2	2.311 (0.319, 16.754)	0.175, 45.6%		–	Very low
**Trial type**							
	RCT	9	2.804 (1.903, 4.132)*	0.998, 0.0%	0.233	0.532, 0.422	Moderate
	Retrospective study	18	2.187 (1.384, 3.454)*	0.045, 39.3%		0.256,0.105	Moderate
**1Y-OS**	**Overall**	30	2.694 (2.050, 3.540)*	0.000, 54.6%^#^		0.035, 0.045^¶^	Very low
**TACE agent classify**							
	Adriamycin+platinum	10	2.186 (1.352, 3.534)*	0.007, 60.5%^#^	0.703	0.032, 0.019^¶^	Very low
	Adriamycin+platinum+fluorouracin	7	2.123 (1.385, 3.253)*	0.314, 15.2%		0.536,0.746	Low
	Platinum+fluorouracin	1	3.000 (0.993, 9.067)	–		–	Very low
	Platinum+raltitrexed	3	7.455 (3.524, 15.772)*	0.251, 27.6%		0.296, 0.058	Very low
	Adriamycin+fluoroura	1	2.667 (0.648, 10.972)	–		–	Very low
	Adriamycin	5	4.055 (2.485, 6.618)*	0.213, 31.3%		0.734, 0.645	Moderate
	Fluorouracil	1	1.833 (0.616, 5.453)	–		–	Very low
	No mention	2	1.273 (0.581, 2.793)	0.617, 0.0%		–	Very low
**Apatinib dosage**							
	500 mg/day	21	3.046 (2.162, 4.293)*	0.000, 61.3%^#^	0.111	0.103, 0.043	Low
	>500 mg/day	8	2.159 (1.468, 3.174)*	0.394, 4.7%		0.108,0.274	Moderate
	<500 mg/day	1	0.893(0.311, 2.561)	–		–	Very low
**Trial type**							
	RCT	9	2.328(1.624, 3.336)*	0.966, 0.0%	0.608	0.095, 0.111	Moderate
	Retrospective study	21	2.870(1.983, 4.152)*	0.000, 67.4%^#^		0.061,0.074	Low
**Progression-free survival (PFS)**							
**6M-PFS**	**Overall**	17	2.783 (1.292, 5.996)*	0.000, 85.6%^#^		0.893, 0.310	Low
TACE agent classify							
	Adriamycin+platinum	8	1.593 (0.484, 5.240)	0.000, 85.7%^#^	0.154	0.072,0.094	Low
	Adriamycin+platinum+fluorouracin	1	2.714 (0.494, 14.901)	–		–	Very low
	Platinum+raltitrexed	1	16.000 (5.443, 47.035)*	–		–	Very low
	Adriamycin	5	4.194 (1.721, 10.221)*	0.023, 64.6%^#^		0.806, 0.367	Low
	No mention	2	4.105 (0.694, 24.264)	0.067, 70.1%^#^		–	Very low
**Apatinib dosage**							
	500 mg/day	12	3.615 (1.597, 8.183)*	0.000, 83.5%^#^	0.167	0.493, 0.144	Low
	>500 mg/day	2	0.736 (0.063, 8.558)	0.025, 80.2%^#^		–	Very low
	<500 mg/day	2	2.115( 0.040, 112.511)	0.000, 96.2%^#^		–	Very low
**Trial type**							
	RCT	4	2.171 (0.148, 31.785)	0.000, 91.4%^#^	0.788	0.602, 0.626	Low
	Retrospective study	13	2.926 (1.291, 6.627)*	0.000, 85.6%^#^		0.393,0.215	Very low
**1Y-PFS**	**Overall**	17	3.837 (2.236, 6.583)*	0.000, 69.3%^#^		0.434, 0.823	Low
TACE agent classify							
	Adriamycin+platinum	7	2.265 (0.783, 6.555)	0.000, 78.7%^#^	0.820	0.881,0.311	Low
	Adriamycin+platinum+fluorouracin	1	3.143 (1.120, 8.822)*	–		–	Very low
	Platinum+raltitrexed	1	10.872 (2.906, 40.673)*	–		–	Very low
	Adriamycin	6	6.528 (2.852, 14.944)*	0.012, 66.1%^#^		0.573,0.231	Low
	No mention	2	1.941 (0.600, 6.287)	0.231, 30.2%		–	Very low
**Apatinib dosage**							
	500 mg/day	13	4.291 (3.126, 5.889)*	0.558, 0.0%	0.634	0.143, 0.416	Moderate
	>500 mg/day	2	1.039 (0.113, 9.554)	0.006, 86.9%^#^		–	Very low
	<500 mg/day	2	1.839 (0.000, 8073.995)	0.000, 96.0%^#^		–	Very low
**Trial type**							
	RCT	4	4.719 (0.673, 33.070)	0.000, 90.5%^#^	0.810	0.497,0.562	Low
	Retrospective study	13	3.821 (2.421, 6.031)*	0.052, 42.6%		0.625,0.373	Moderate
**AFP**	**Overall**	8	−2.628 (−3.959, −1.296)*	0.000, 97.4%^#^		0.108,0.006^¶^	Very low
**MMP9**	**Overall**	6	1.650 (−0.370, 3.671)	0.000, 98.2%^#^		0.260,0.130	Low
**VEGF**	**Overall**	8	−1.317 (−2.897, 0.263)	0.000, 97.9%^#^		0.902,0.873	Low

^*^*Significant differences, ^#^Substantial heterogeneity, ^¶^Publication bias.

For 6M-OS, significant differences could be found in these subgroups, namely, overall (2.311, 1.744–3.062), adriamycin+platinum (2.219, 1.376–3.579), adriamycin+platinum+fluorouracin (2.666, 1.565–4.540), and platinum+raltitrexed (11.748, 2.154–64.078) subgroups with low heterogeneity (*I*^2 =^ 0%–38.7%). In the apatinib dosage subgroups, significance could be found in 500 mg/day (2.305, 1.604–3.311) and >500 mg/day (2.676, 1.587–4.511). In trial type subgroups, significant difference could also be found in both RCT (2.804, 1.903–4.132) and retrospective study (2.187, 1.384–3.454). In evaluating 1Y-OS, significant differences were found in overall (2.694, 2.050–3.540), adriamycin+platinum (2.186, 1.352–3.534), adriamycin+platinum+fluorouracin (2.123, 1.385–3.253), platinum+raltitrexed (7.455, 3.524–15.772), and adriamycin (4.055, 2.485–6.618) subgroups with moderate heterogeneity(*I*^2^ = 15.2%–60.5%). Furthermore, significant differences can be found in 500 and >500 mg/day subgroups and RCT and retrospective study subgroups. Meta-regression did not reveal the source of heterogeneity with few publication biases and low to moderate grades (**Table 2**).

For 6M-PFS, significant outcomes could be found in the overall group and TACE agent classification including adriamycin+platinum+fluorouracin, platinum+raltitrexed, and adriamycin subgroups, apatinib dosage of 500 mg/day group, and trial type as retrospective study group. In evaluating 1Y-PFS, significant differences could be found in TACE agent classification including adriamycin+platinum +fluorouracin, platinum+raltitrexed, and adriamycin subgroups, apatinib dosage of 500 mg/day group, and trial type as retrospective study group. In addition, TACE+A treatment could decease the expression of AFP (−2.628, −3.959 to −1.296) with substantial heterogeneity (*I*^2^ = 97.4). No significant results were found in decreasing MMP9 and VEGF value (**Table 2**).

In general, TACE + A treatment is beneficial in efficacy compared with TACE treatment alone. Even if there is no significant difference in meta-regression, we can notice that the groups with positive results and more original literatures were basically adriamycin+platinum, adriamycin+platinum+fluorouracin, platinum+fluorouracin, and adriamycin from the results. Therefore, we conducted a follow-up network meta-analysis to confirm which agent is the most suitable to be combined with apatinib for TACE treatment.

### Network Meta-Analysis for Confirming the Most Suitable Agent Combined With Apatinib for TACE Treatment

To determine the most suitable agent type of TACE, the first-time network meta-analysis was used to determine the ranking of adriamycin, platinum, and fluorouracin in the efficacy TR, 6M-OS, and 1Y-OS. From the first-time network meta-analysis, we noticed that adriamycin always ranked first compared with the other agents, followed by platinum and fluorouracin, with very little difference among them (**Table 3**). Based on the above results, we still do not know which agents should be selected as TACE treatment agent when combined with apatinib. Therefore, we need to conduct the next network meta-analysis to determine this.

**Table 3:** Efficacy of TACE+apatinib versus TACE in treatment response and overall survival, which was subgroup analyzed by classification of TACE agents.

Outcome	Subgroups	Included RCTs	OR(95%CI) from pairwise meta-analysis	*p*, *I*^2^	*p* from meta-regression	OR (95%CI) from network meta-analysis	Rank
**Treatment response (TR)**	Adriamycin	14	2.514 (1.919, 3.293)*	0.622, 0.0%	0.342	2.42 (1.86, 3.15)*	1
	Platinum	11	2.520 (1.892, 3.358)*	0.886, 0.0%	0.513	2.44 (1.85, 3.22)*	2
	Fluorouracin	6	2.168 (1.449, 3.242)*	0.772, 0.0%	0.508	2.33 (1.63,3.32)*	3
**6M-OS**	Adriamycin	9	2.804 (1.903, 4.132)*	0.998, 0.0%	–	2.82 (1.92,4.14)*	1
	Platinum	7	2.927 (1.898, 4.513)*	0.992, 0.0%	0.676	2.88 (1.89,4.39)*	2
	Fluorouracin	5	3.113 (1.683, 5.758)*	0.995, 0.0%	0.681	2.99 (1.67,5.34)*	3
**1Y-OS**	Adriamycin	9	2.328(1.624, 3.336)*	0.966, 0.0%	–	2.37 (1.66,3.99)*	1
	Platinum	7	2.343(1.590, 3.455)*	0.891, 0.0%	0.931	2.39 (1.64,3.50)*	2
	Fluorouracin	5	2.330(1.453, 3.738)*	0.818, 0.0%	0.994	2.39 (1.54,3.72)*	3

*Significant differences.

**Figure 2:**
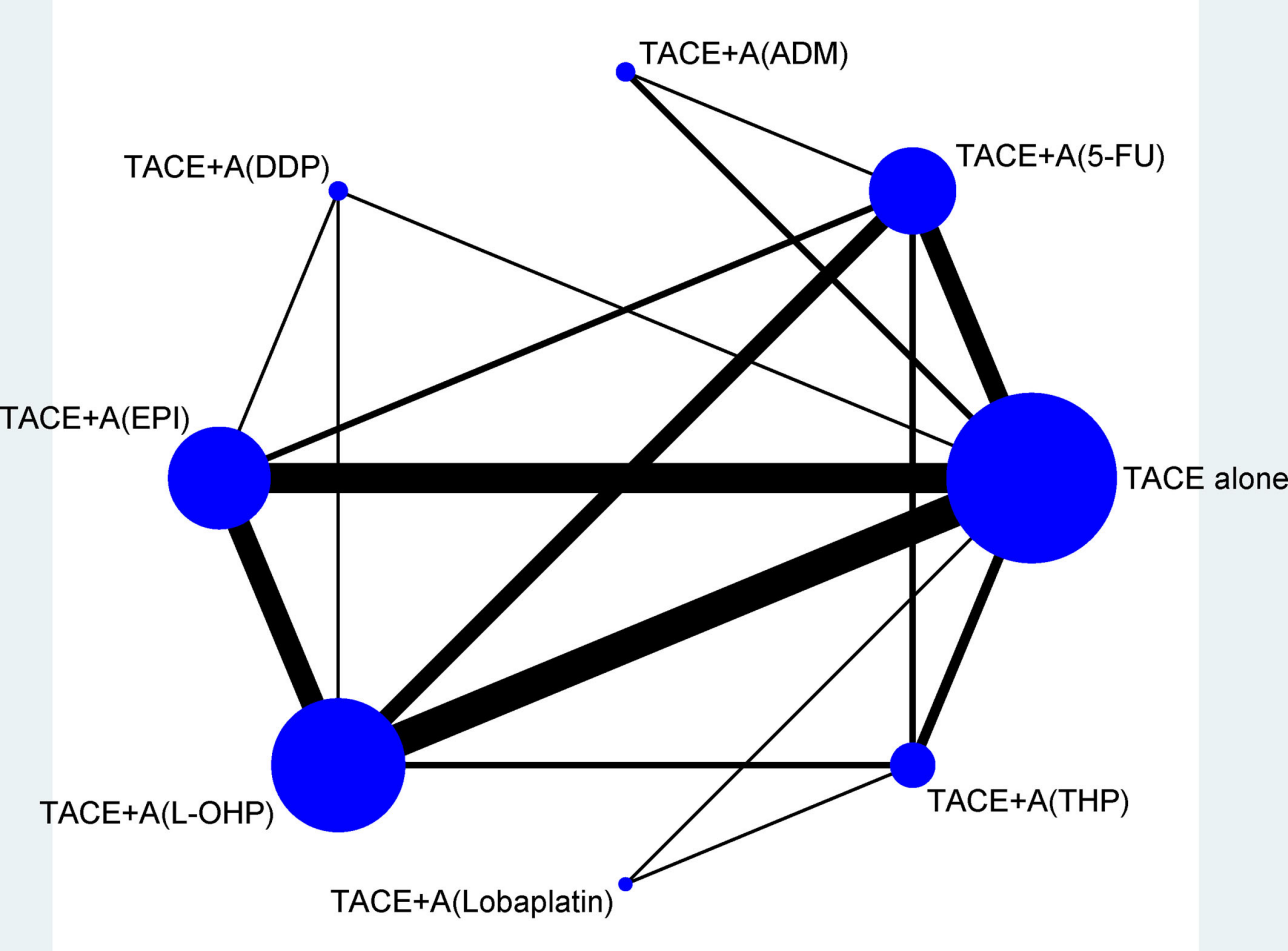
Network of eligible efficacy comparisons of TACE + apatinib versus TACE in treatment response. The width of the lines is proportional to the number of studies comparing every pair of direct comparisons of treatment agents in TACE, and the size of each node is proportional to the number of treatment participants. 5-FU, 5-fluorouracil; A, apatinib; ADM, doxorubicin; DDP, cisplatin; EPI, epirubicin; L-OHP, oxaliplatin; TACE, transarterial chemoembolization; THP, pirarubicin.

**Figure 3:**
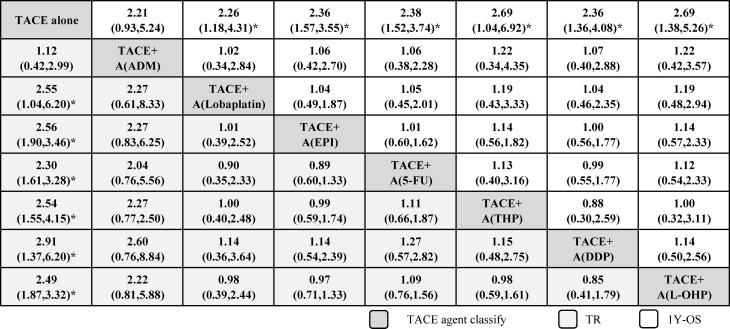
League tables of efficacy of TACE+apatinib versus TACE in treatment response and 1-year OS. Comparisons between treatment agents should be read from left to right, and the estimates in the cell are common between the column-defining treatment and the row-defining treatment. For efficacy of treatment response and 1-year OS, an OR more than 1 favors the column-defining treatment. To obtain ORs for comparisons in the opposing direction, reciprocals should be taken. 5-FU, 5-fluorouracil; A, apatinib; ADM, doxorubicin; DDP, cisplatin; EPI, epirubicin; L-OHP, oxaliplatin; RTACE, transarterial chemoembolization; THP, pirarubicin. *Significant results are in bold.

**Figure 2** shows the network plots for TR, and the node represents an agent used in TACE therapy. **Figure 3** shows the league tables for the network, which estimates all comparisons for TR and 1Y-OS. In evaluating TR, compared with TACE treatment alone, TACE(L-OHP, oxaliplatin)+A ranked first with significant difference (2.49, 1.87–3.32), followed by TACE(DDP, cisplatin)+A (2.91, 1.37–6.20), TACE(THP, pirarubicin)+A (2.54, 1.55–4.15), TACE(5-FU, 5-fluorouracil)+A (2.30, 1.61–3.28), TACE(EPI, epirubicin)+A (2.56, 1.90–3.46), TACE(Lobaplatin)+A (2.55, 1.04–6.20), and TACE(ADM)+A. No significant differences were detected between comparisons. For 1Y-OS, compared with TACE alone, TACE(L-OHP, oxaliplatin)+A ranked first (2.69, 1.38–5.26), followed by TACE(THP, pirarubicin)+A (2.69, 1.04–6.92), TACE(5-FU, 5-fluorouracil)+A (2.38, 1.52–3.74), TACE(DDP, cisplatin)+A (2.36, 1.36–4.08), TACE(EPI, epirubicin)+A(2.36, 1.57–3.55), TACE(Lobaplatin)+A (2.26, 1.18–4.31), and TACE(ADM)+A. In summary, the most suitable agent combined with apatinib for TACE treatment may appear in the L-OHP, DDP, THP, EPI, and 5-FU subgroups.

### Pairwise Meta-Analysis Outcomes of TACE+Apatinib Versus TACE Therapy in Safety

For the safety results, we selected only the adriamycin+platinum+fluorouracil and adriamycin+platinum subgroups with better efficacy, and took hypertension, hand–foot syndrome, fatigue, fever, nausea–vomiting, and diarrhea index into consideration. We could notice that the TACE+A group could significantly increase risk of hypertension and hand–foot syndrome in both adriamycin+platinum+fluorouracil and adriamycin+platinum subgroups with substantial heterogeneity. The adriamycin+platinum+fluorouracil subgroup could also increase the risk of diarrhea (**Table 4**). Based on these outcomes, we believe that adriamycin+platinum ± fluorouracil combination therapy in TACE was relatively safe and can be applied by paying attention to the prevention of hypertension, hand–foot syndrome, and diarrhea.

**Table 4:** Safety of TACE+apatinib versus TACE in adverse effect, which was analyzed by classification of TACE agents.

Outcome	Subgroups	Included RCTs	OR(95%CI) from pairwise meta-analysis	*p*, *I*^2^	Publication bias	Grade
**Hypertension**	Adriamycin+platinum+fluorouracil	5	25.007 (5.186, 120.575)*	0.002, 76.9%^#^	0.327, 0.044^¶^	Low
	Adriamycin+platinum	10	16.078( 5.551, 46.566)*	0.000, 81.5%^#^	0.074, 0.015^¶^	Low
**Hand-foot syndrome**	Adriamycin+platinum+fluorouracil	6	14.349 (2.271, 90.649)*	0.004, 71.0%^#^	0.851, 0.216	Moderate
	Adriamycin+platinum	9	12.496 (3.123, 50.004)*	0.000, 85.0%^#^	0.037, 0.000^¶^	Low
**Fatigue**	Adriamycin+platinum+fluorouracil	4	1.686( 0.711, 3.995)	0.028, 63.1%	0.050, 0.004^¶^	Moderate
	Adriamycin+platinum	5	1.194 (0.735, 1.939)	0.897, 0.0%	0.174, 0.332	High
**Fever**	Adriamycin+platinum+fluorouracil	4	1.138 (0.690, 1.875)	0.479, 0.0%	0.34, 0.646	High
	Adriamycin+platinum	9	1.086 (0.805, 1.464)	0.997, 0.0%	0.029, 0.023^¶^	Moderate
**Nausea–vomiting**	Adriamycin+platinum+fluorouracil	6	1.445 (0.845, 2.472)	0.954, 0.0%	0.190, 0.756	High
	Adriamycin+platinum	10	1.050 (0.711, 1.550)	0.170, 29.9%	0.655, 0.586	High
**Diarrhea**	Adriamycin+platinum+fluorouracil	7	2.331 (1.194, 4.553)*	0.328, 13.4%	0.099, 0.072	High
	Adriamycin+platinum	10	1.781 (0.859, 3.695)	0.001, 66.6%^#^	0.655, 0.117	Moderate

*Significant differences ^#^Substantial heterogeneity ^¶^Publication bias.

## Discussion

To identify the most suitable agent in TACE combined therapy with apatinib, we identified 45 original studies including 3,876 patients with HCC (**Table 1****;** **Figure 1**). First, from pairwise meta-analysis, we identified that significant outcomes always appear in subgroups of adriamycin+platinum, adriamycin+platinum+fluorouracin, and platinum+fluorouracin, the grouping of which was based on TACE agent classification, apatinib dosage of 500 and >500 mg/day, in both RCT and retrospective studies (**Table 2**). Second, we conducted a follow-up network meta-analysis to confirm the most suitable agent combined with apatinib for TACE treatment; we made a conclusion that significance may appear in the L-OHP, DDP, THP, EPI, and 5-FU subgroups (**Table 3****;** **Figures 2**, **3**). Third, adriamycin+platinum ± fluorouracil combination therapy in TACE was relatively safe (**Table 4**).

This systematic review and network meta-analysis followed the PRISMA guideline and was registered on PROSPERO website. The efficacy outcomes that we made could be proofed with some publications. Cao’s propensity score matching study made concluded that TACE+apatinib treatment could improve the prognosis compared with TACE alone in OS and TTP, etc. TACE+apatinib treatment could inhibit metastasis after TACE procedure with contracted tumor feeding artery for advanced HCCs without metastasis (70). In addition, Li’s research revealed that DEB-TACE plus apatinib achieves prolonged PFS and OS, while similar adverse events occurrence were observed when compared to DEB-TACE alone in huge HCC treatment (71). Moreover, Ju’s research declared that DEB-TACE followed by apatinib is effective and safe in treating BCLC stage C HCC patients, which indicates its role as an acceptable option in HCC management (72). A real-world study design by Wang indicated that TACE plus apatinib-combined therapy (vs. TACE) independently related to the longer OS (hazard ratio: 0.504, *p*=0.001). In TACE plus apatinib combined therapy group, the most frequent adverse events included hand–foot syndrome (95.8%), hypertension (95.8%), fatigue (90.8%), albuminuria (85.7%), anorexia (79.0%), diarrhea (66.4%), myelosuppression (58.8%), nausea/vomiting (49.6%), and abdominal pain (39.5%); morever, no grade 4 adverse events and treatment-related death occurred (73). The above studies were similar to the results of our network meta-analysis, which proves that TACE + A treatment has advantages in both efficacy and safety.

We made the conclusion that adriamycin+platinum ± fluorouracil combination therapy in TACE+A was more beneficial in patients with HCC, and the results can also be demonstrated by some publications. Zhao’s meta-analysis concluded that TACE with platinum revealed similar clinical efficacy compared with anthracyclines (74). Besides, platinum agent miriplatin became standard medicines in addition to anthracyclines in TACE (75). Moreover, TACE with doxorubicin-eluting beads was effective in patients with Barcelona clinic liver cancer stage B HCC (76). Furthermore, Lammer’s research made a conclusion that TACE with DC Bead and doxorubicin is safe and effective in the treatment of HCC and offers a benefit to patients with more advanced disease (77). In summary, adriamycin+platinum ± fluorouracil combination therapy in TACE+A is acceptable.

There are also some limitations in our research. First, the originally studies that we included were mostly done in China, which may bring geographic heterogeneity in our studies. Only 17 of 45 studies were RCTs, which may have a certain effect on the quality of outcomes. Second, for the nodes of network meta-analysis, there may be duplication. For example, if an original article is treated with three agents, there will be the same data in all three nodes. Third, the SUCRA score in subgroups we obtained from network meta-analysis was not very different from each other.

Previous meta-analysis only suggest the use of TACE+A (78–80) in HCC patients, our research is the first network meta-analysis that provided the most suitable agent in TACE combined therapy with apatinib. We concluded that adriamycin+platinum ± fluorouracil combination therapy in TACE+A is efficacious and safe. For novel molecular targeted drugs related to HCC molecular targeted therapy, the current clinical trials are mainly the comparison between various drugs and sorafenib (81, 82). Further research should pay more attention to the agents in TACE, with a larger sample size and a multicenter and better study design.

In conclusion, the most suitable agents in TACE combined with apatinib are adriamycin+platinum ± fluorouracil combination therapy, especially THP and EPI as adriamycin, L-OHP and DDP as platinum, and 5-FU as fluorouracil. Therefore, we recommend that patients with HCC who require TACE treatment with apatinib should be treated with the above three type agents.

## Data Availability Statement

The original contributions presented in the study are included in the article/**Supplementary Material**. Further inquiries can be directed to the corresponding author.

## Author Contributions

The authors declare that the research was conducted in the absence of any commercial or financial relationships that could be construed as a potential conflict of interest.

## Funding

This study was supported by the Doctoral Scientific Research Foundation of Liaoning Province (nos. 2020-BS-125 and 2021-BS-135).

## Conflict of Interest

The authors declare that the research was conducted in the absence of any commercial or financial relationships that could be construed as a potential conflict of interest.

## Publisher’s Note

All claims expressed in this article are solely those of the authors and do not necessarily represent those of their affiliated organizations, or those of the publisher, the editors and the reviewers. Any product that may be evaluated in this article, or claim that may be made by its manufacturer, is not guaranteed or endorsed by the publisher.

## Supplementary Material

Supplementary Table 1 Study Characteristics.

Supplementary Table 2 Risk of Bias Assessments by NOS scale.

Supplementary Figure 1 Risk of bias summary Cochrane Collaboration’s tool.

Supplementary Figure 2 Inconsistency plot of included studies with treatment response.

Supplementary Figure 3 Network funnel plot of included studies with treatment response.
