# Supplementary material for: What Is the Most Suitable Agent Combined With Apatinib for Transarterial Chemoembolization Treatment in Advanced Hepatocellular Carcinoma Patients? A Systematic Review and Network Meta-analysis
Source: Front Oncol. 2022 May 25;12:887332. doi: 10.3389/fonc.2022.887332 (PMC9174538; doi:10.3389/fonc.2022.887332)
Supplement: Supplementary Table 1 — Study Characteristics. [file Table_1.doc]

**Table S1 Baseline Characteristic of included studies**

| **Publication, year** | **Trial type** | **Sample size(gender)** | **Age** | **HBV [Infection](../../../../D:/Program%20Files%20(x86)/Youdao/Dict/8.9.8.0/resultui/html/index.html#/javascript:;)** | **TNM stage(I-II/III-IV)** | **Tumor size(<5/≥5)** | **BCLC stage(B/C)** | **Child-Pugh**  **Classification(A/B-C)** | **ECOG score (0-1/2)** | **Cycles of TACE** | **Intervention arm** | **Control arm** | **Apatinib dosage** | **TACE agent** |
| --- | --- | --- | --- | --- | --- | --- | --- | --- | --- | --- | --- | --- | --- | --- |
| Qiu Z,2021^[1]^ | Retrospective study | I:86(78/8)  C:115(104/11) | - | I:74/12  C:105/10 |  | I:33/53  C:34/81 |  | I:70/16  C:96/19 |  | I:3.2±1.7  C:3.5±1.9 | TACE-apatinib | TACE-sorafenib | 500mg, 1/day | 30–50 mg of EPI , 30–50 mg of lobaplatin, and 5–15 mL of lipiodol |
| Zhang H,2021^[2]^ | Retrospective study | I:29(24/5)  I:29(25/4) | I:50.9±8.9  C:53.4±8.3 |  |  | I:15/14  C:16/13 | I:16/13  C:11/18 | I:23/6  C:19/10 |  |  | TACE-apatinib | TACE | 500mg, 2/day | 5-FU 500g/m2, DDP 40mg/m2, EPI 40mg/m2, liquified lipiodol 10-30ml |
| Sun Z,2022^[3]^ | Retrospective study | I:21(19/2)  C:60(49/11) | I:52.68±11.06  C:62.42±9.69 |  |  |  | I:8/13  C:27/33 |  |  |  | TACE-apatinib | TACE | 500mg, 1/day | No mention |
| Cai Z,2020^[4]^ | RCT | I:50(27/23)  C:50(26/24) | I:46.57±4.23  C:46.28±4.21 |  |  |  |  |  |  |  | TACE-apatinib | TACE | 500mg, 1/day | L-OHP 100mg, 5-Fu 1g |
| Chen L,2020^[5]^ | Retrospective study | I:83(76/7)  C:82(78/15) | I:50.8±9.6  C:53.6±10.8 | I:71/12  C:71/11 |  |  |  |  | I:8/75  C:6/76 |  | TACE-apatinib | TACE | 500mg, 1/day | 5–15mL of emulsion containing 10–20 mg of ADM hydrochloride were mixed with 5–10mL of lipiodol |
| Fan Z,2020^[6]^ | Retrospective study | I:35(23/12)  C:33(23/10) | I:51.97±13.30  C:53.82±12.29 | I:14/7  C:8/11 |  |  | I:19/16  C:18/15 | I:15/20  C:17/16 | I:12/23  C:14/19 | I:4.63±1.93  C:4.61±2.19 | TACE-apatinib | TACE-sorafenib | 750mg, 1/day | THP 20mg, 5-FU 1000mg, L-OHP 150-200mg |
| Gu H,2020^[7]^ | RCT | I:40(19/21)  C:40(22/18) | I:53(31-68)  C:51(33-67) | I:28/12  C:30/10 |  | I:31/9  C:29/11 | I:36/4  C:33/7 |  | I:33/7  C:35/5 |  | TACE-apatinib | TACE | 500mg, 1/day | Lobaplatin 50mg, EPI 50mg, mixed with 5–10 mL of ethiodized oil |
| Han X,2020^[8]^ | RCT | I:50(41/9)  C:50(40/10) | I:56.90±4.72  C:56.84±4.67 |  |  |  | I:43/7  C:44/6 | I:39/11  C:38/12 |  |  | TACE-apatinib | TACE | 500mg, 1/day | EPI 30 mg, L-OHP 50 mg, iodized oil 10~20 M L |
| He Y,2020^[9]^ | RCT | I:50(28/22)  C:50(30/20) | I:62.33±7.18  C:63.23±7.20 |  |  |  |  |  |  |  | TACE-apatinib | TACE | 750mg, 1/day | EPI 60mg, DDP 60mg, and 5-FU 750mg |
| He YK,2020^[10]^ | RCT | I:52(33/19)  C:52(38/14) | I:60.02±11.45  C:59.64±10.15 |  |  |  |  |  |  |  | TACE-apatinib | TACE | 500mg, 1/day | L-OHP 80-100mg + EPI 10-20mg + 5-FU 0.5-1.0g |
| Huang W,2020^[11]^ | RCT | I:36(21/15)  C:36(20/16) | I:51.06±2.79  C:52.18±2.55 |  |  |  |  | I:22/14  C:23/13 |  |  | TACE-apatinib | TACE | 500mg, 1/day | THP 40mg, Lobaplatin 10-20mg, maxed with [Lipiodol](../../../../D:/Program%20Files%20(x86)/Youdao/Dict/8.9.8.0/resultui/html/index.html#/javascript:;) |
| Kan X,2020^[12]^ | Retrospective study | I:90(77/13)  C:90(78/12) | I:52.7 ± 9.7  C:53.1 ± 10.1 | I:80/10  C:79/11 |  |  |  | I:77/13  C:79/11 | I:74/16  C:75/15 |  | TACE-apatinib | TACE | 500mg, 1/day | 10–20 ml of lipiodol was mixed with 20–40 mg of ADM |
| Li F,2020^[13]^ | Retrospective study | I:71(46/25)  C:81(52/29) | I:67.3±7.4  C:66.8±6.9 |  | I:53/18  C:61/20 |  |  | I:51/20  C:58/23 |  | I:2.53±0.53  C:3.34±0.72 | TACE-apatinib | TACE | 500mg, 1/day | 50 mg L-OHP +30 mg EPI 5 ~ 25 mL iodized oil |
| Shuanggang C,2020^[14]^ | Retrospective study | I:20(19/1)  C:20(17/3) |  | I:18/2  C:18/2 |  | I:2/18  C:2/18 |  | I:19/1  C:17/3 |  |  | TACE‑MWA‑apatinib (n=20) | TACE | 500mg, 1/day | lipiodol: lobaplatin: THP = 5–15 ml: 30–50 mg: 30–50 mg |
| Sun T,2020^[15]^ | Retrospective study | I:27(24/3)  C:31(21/10) | I:55.56 ± 5.2  C:55.56 ± 5.2 | I:25/2  C:28/3 |  |  |  | I:21/6  C:23/8 | I:21/6  C:25/6 |  | TACE-apatinib | TACE | 500mg, 1/day | 2–20 mL of lipiodol was mixed with 20–40 mg ADM hydrochloride |
| Wu H,2020^[16]^ | Retrospective study | I:36(24/12)  C:28(17/11) | I:61.9±9.35  C:60.54±8.34 | I:26/10  C:20/8 |  |  | I:24/12  C:19/9 | I:25/13  C:18/10 |  | I:3.24±1.02  C:5.34±1.45 | TACE-apatinib | TACE | 500mg, 1/day | Lopaplatin 50mg, THP 40mg, mixed with lipiodol |
| Wu P,2020^[17]^ | Retrospective study | I(a):30(13/17)  I(b):30(15/15)  C:30(16/14) | I(a):57.6±8.23  I(b):56.54±6.37  C:56.76±6.18 |  |  |  | I(a):26/4  I(b):27/3  C:26/4 | I(a):23/7  I(b):22/8  C:20/10 |  | I(a):3.56±1.12  I(b):2.64±1.21  C:3.14±1.57 | TACE-apatinib | TACE | I(a):550mg, 1/day  I(b):300mg, 1/day | Lobaplatin 50mg , [Raltitrexed](../../../../D:/Program%20Files%20(x86)/Youdao/Dict/8.9.8.0/resultui/html/index.html#/javascript:;) 4mg |
| Xu B,2020^[18]^ | Retrospective study | I:24(20/4)  C:24(19/5) | I:56.3±10.4  C:59.0±9.8 |  |  |  | I:13/11  C:15/9 | I:17/7  C:15/9 | I:18/6  C:19/5 | I:3.25±0.38  C:2.83±0.27 | TACE-apatinib | TACE | 500mg, 1/day | EPI 30mg, mixed with lipiodol |
| Xu H,2020^[19]^ | Retrospective study | I:31(19/12)  C:30(20/10) | I:56.39±7.99  C:54.57±7.87 | I:22/9  C:18/12 |  |  |  | I:10/21  C:8/22 |  |  | TACE-apatinib | TACE | 500mg, 1/day | 5-FU, L-OHP, mixed with lipiodol |
| Yang Y,2020^[20]^ | Retrospective study | I:30(17/13)  C:30(20/10) | I:48.75±12.28  C:51.03±11.67 | I:24/6  C:26/4 |  |  | I:16/14  C:18/12 | I:11/19  C:13/17 | I:18/12  C:17/13 |  | TACE-apatinib | TACE | 500mg, 1/day | Lobaplatin 30mg, EPI 50mg, FT-207 1g |
| Cao F,2019^[21]^ | Retrospective study | I:42(29/13)  C:56(38/18) | I:58.31±8.73  C:57.80±8.03 | I:36/6  C:47/9 |  |  | I:9/33  C:13/43 | I:32/10  C:45/11 | I:33/9  C:46/10 | I:7.8±3.8  C:7.6±3.4 | TACE-apatinib | TACE | 500mg, 1/day | [Raltitrexed](../../../../D:/Program%20Files%20(x86)/Youdao/Dict/8.9.8.0/resultui/html/index.html#/javascript:;) 4mg, Lobaplatin 40mg |
| Cui H,2019^[22]^ | Retrospective study | I:25(17/8)  C:25(16/9) | I:51.6(31.2,71.8)  C:52.9(29.7,77.6) | I:18/7  C:20/5 | I:25/0  C:25/0 |  |  | I:17/8  C:19/6 |  |  | TACE-apatinib | TACE | 500mg, 1/day | ADM 30mg or DDP 100mg |
| Fan W,2019^[23]^ | Retrospective study | I:85(68/17)  C:103(71/32) | I:49 (17-71)  C:50 (19-80) | I:68/15  C:78/25 |  | I:58/27  C:80/23 |  | I:73/12  C:87/16 | I:67/18  C:90/13 |  | TACE-apatinib | TACE | 500mg, 1/day | CBP 300mg, EPI 50mg, mixed with lipiodol 5ml |
| Li W,2019^[24]^ | Retrospective study | I:30(23/7)  C:30(22/8) | I:51.6±9.8  C:55.2±12.1 |  |  |  | I:24/6  C:23/7 | I:23/7  C:25/5 |  | I:3.2±0.8  C:3.6±1.1 | TACE-apatinib | TACE | 500mg, 1/day | L-OHP, 5-FU, EPI, Iodinated Oil |
| Pu J,2019^[25]^ | RCT | I:45  C:45 | 54.8±7.6 |  |  |  |  |  |  |  | TACE-apatinib | TACE | 250mg, 2/day | DDP 150mg + L-OHP 50mg + EPI 30mg, Iodinated Oil |
| Qiao X,2019^[26]^ | Retrospective study | I:108(59/49)  C:102(56/46) | I:56.5±6.3  C:58.2±5.4 |  |  |  | I:96/12  C:77/25 | I:81/27  C:73/29 |  | I:3.1±1.3  C:2.8±2.0 | TACE-apatinib | TACE | 250mg, 2/day | EPI, Iodinated Oil |
| Qiu Z(a),2019^[27]^ | Retrospective study | I:42(41/1)  C:83(73/10) |  |  |  | I:17/25  C:33/50 | I:9/33  C:29/54 | I:36/6  C:75/8 |  |  | TACE-apatinib | TACE | 500mg, 1/day | 5–15 mL of lipiodol, 30–50 mg THP and 30–50 mg lobaplatin |
| Qiu Z(b),2019^[27]^ | Retrospective study | I:29(28/1)  C:29(27/2) |  |  |  | I:12/17  C:14/15 | I:7/22  C:11/18 | I:25/4  C:26/3 |  |  | TACE-apatinib | TACE | 500mg, 1/day | 5–15 mL of lipiodol, 30–50 mg THP and 30–50 mg lobaplatin |
| Shen R,2019^[28]^ | Retrospective study | I:38(23/15)  C:51(31/20) | I:59.13±7.49  C:58.47±7.26 |  |  |  | I:33/5  C:42/9 | I:22/16  C:30/21 |  |  | TACE-apatinib | TACE | 500mg, 1/day | L-OHP 100mg + EPI 20mg + iodized oil 5 ~ 20ml |
| Weng Z,2019^[29]^ | RCT | I:35(26/9)  C:35(25/10) | I:51.77±2.90  C:52.26±3.19 |  |  |  | I:24/11  C:23/12 |  |  |  | TACE-apatinib | TACE | 500mg, 1/day | Iodinated oil, L-OHP, 5-FU and THP |
| Wu F,2019^[30]^ | Retrospective study | I:28(18/10)  C:31(23/8) | I:57.2±7.0  C:58.0±7.1 |  |  |  |  | I:25/3  C:26/5 |  |  | TACE-apatinib | TACE | 500mg, 1/day | L-OHP, and 5-FU , followed by superliquefied iodinated oil and THP suspension |
| Wu Y,2019^[31]^ | RCT | I:42(27/15)  C:41(25/16) | I:55.43±3.69  C:55.16±3.48 |  |  |  |  |  |  |  | TACE-apatinib | TACE | 500mg, 1/day | EPI 40mg |
| Xiao Q,2019^[32]^ | Retrospective study | I:38(30/8)  C:52(38/14) | I:62.2±13.2  C:58.1±10.1 |  |  |  |  | I:20/18  C:27/25 |  |  | TACE-apatinib | TACE | 250mg, 1/day | L-OHP 0.1g+ 5-FU 0.5g, EPI 20mg, iodized oil |
| Xiong J,2019^[33]^ | Retrospective study | I:20(16/4)  C:25(18/7) | I:53  C:56 |  |  | I:8/12  C:14/11 |  | I:14/6  C:18/7 |  |  | TACE-apatinib | TACE | 500mg, 1/day | FT207 40mg/m2 |
| Yang Q,2019^[34]^ | RCT | I:23(12/11)  C:23(12/11) | I:45.03±5.62  C:45.53±5.85 |  |  |  | I:9/14  C:7/16 |  |  |  | TACE-apatinib | TACE | 500mg, 1/day | ADM |
| Yang Z,2019^[35]^ | Retrospective study | I:25(20/5)  C:22(18/4) |  | I:18/7  C:14/8 |  |  | I:10/15  C:10/12 |  | I:19/6  C:22/9 |  | TACE-apatinib | TACE | 500mg, 1/day | No mention |
| Zhu Y,2019^[36]^ | RCT | I:44(32/12)  C:44(34/10) |  |  |  |  |  | I:38/6  C:36/8 |  |  | TACE-apatinib | TACE | 500mg, 1/day | EPI, L-OHP, iodized oil |
| Zhu Y,2019^[37]^ | Retrospective study | I:25(20/5)  C:25(17/8) | I:58.34±5.67  C:59.22±5.17 |  |  |  |  |  |  |  | TACE-apatinib | TACE | 500mg, 1/day | No mention |
| Chen S,2019^[38]^ | Retrospective study | I:27(23/4)  C:53(43/10) | I:45.8±11.0  C:54.4 ± 11.9 | I:26/1  C:49/4 |  |  |  | I:21/6  C:48/5 |  | I:2.19±1.04  C:2.34±3.99 | TACE-apatinib | TACE | 500mg, 1/day | No mention |
| He F,2018^[39]^ | RCT | I:50(24/26)  C:50(28/22) | I:53.1±1.5  C:52.7±1.5 |  |  |  |  |  |  |  | TACE-apatinib | TACE | 400mg, 1/day | Lobaplatin, EPI, iodized oil |
| Huang R,2018^[40]^ | Retrospective study | 30/30(45/15) | 54(26-76) |  |  |  | 47/13 |  |  | I:3.2±0.8  C:3.2±1.4 | TACE-apatinib | TACE | 500mg, 1/day | 5-FU, iodized oil |
| Wu J,2018^[41]^ | Retrospective study | I:28(20/8)  C:28(18/10) | I:57.7±8.3  C:56.4±4.2 |  |  |  | I:25/3  C:21/7 | I:21/7  C:20/8 |  | I:3.23±1.2  C:2.67±2.1 | TACE-apatinib | TACE | 500mg, 1/day | EPI |
| Zeng G,2018^[42]^ | RCT | I:38(18/20)  C:38(21/17) | I:56.4±8.8  C:58.82±7.5 |  |  |  | I:13/25  C:16/22 |  |  |  | TACE-apatinib | TACE | 850mg, 1/day | EPI 30-60 mg + L-OHP 50 mg was fully mixed and emulsified with iodized oil 10-20 mL |
| Jin X,2018^[43]^ | RCT | I:22  C:22 | 58.5(34-79) | 35/7 |  |  | 37/7 |  |  |  | TACE-apatinib | TACE | 850mg, 1/day | EPI 30mg+ L-OHP 50mg+ iodinated oil 5-25ml |
| Li W,2018^[44]^ | RCT | I:20(10/10)  C:20(12/8) | I:43.9±5.1  C:43.9±5.1 |  |  |  | I:13/7  C:15/5 |  |  |  | TACE-apatinib | TACE | 850mg, 1/day | 5-FU, ADM, iodized oil |
| Lu W,2018^[45]^ | Retrospective study | I:20(16/4)  C:22(17/5) | I:56.1 ± 10.79  C:58.9 ±9.38 |  |  |  | I:18/2  C:19/3 | I:17/3  C:18/4 |  |  | TACE-apatinib | TACE | 500mg, 1/day | 30 mg of EPI powder, 50 mg of L-OHP powder, 10–20 ml of lipiodol and 10–20 ml of iodine contrast agent |

5-FU, 5-fluorouracil; ADM, doxorubicin; DDP, cisplatin; EPI, epirubicin; FT207, tegafur; L-OHP, oxaliplatin; RCT, [randomized](../../../../D:/Program%20Files%20(x86)/Youdao/Dict/8.9.8.0/resultui/html/index.html#/javascript:;) [controlled](../../../../D:/Program%20Files%20(x86)/Youdao/Dict/8.9.8.0/resultui/html/index.html#/javascript:;) [trial](../../../../D:/Program%20Files%20(x86)/Youdao/Dict/8.9.8.0/resultui/html/index.html#/javascript:;); TACE, Transarterial chemoembolization; THP, pirarubicin.
