# Supplementary material for: What Is the Most Suitable Agent Combined With Apatinib for Transarterial Chemoembolization Treatment in Advanced Hepatocellular Carcinoma Patients? A Systematic Review and Network Meta-analysis
Source: Front Oncol. 2022 May 25;12:887332. doi: 10.3389/fonc.2022.887332 (PMC9174538; doi:10.3389/fonc.2022.887332)
Supplement: Supplementary Table 2 — Risk of Bias Assessments by NOS scale. [file Table_2.doc]

**Table S2 Risk of Bias Assessment using the Newcastle-Ottawa Scale for Retrospective Studies**

| **Publication(year)** | **Is the case definition adequate** | **Representativeness of the cases** | **Selection of Controls** | **Definition of Controls** | **Comparability of cases and controls (/2)** | **Ascertainment of exposure** | **Same method of ascertainment for cases and controls** | **Non-Response rate** | **Overall rating and TOTAL SCORE / 10** |
| --- | --- | --- | --- | --- | --- | --- | --- | --- | --- |
| Qiu Z,2021^[25]^ | 1 | 1 | 0 | 1 | 1 | 1 | 1 | 1 | 7 |
| Zhang H,2021^[26]^ | 1 | 1 | 0 | 1 | 1 | 1 | 1 | 1 | 7 |
| Sun Z,2022^[27]^ | 1 | 1 | 0 | 1 | 1 | 1 | 1 | 1 | 7 |
| Chen L,2020^[29]^ | 1 | 1 | 0 | 1 | 2 | 1 | 1 | 1 | 8 |
| Fan Z,2020^[30]^ | 1 | 1 | 0 | 1 | 1 | 1 | 1 | 1 | 7 |
| Huang W,2020^[36]^ | 1 | 1 | 0 | 1 | 2 | 1 | 1 | 1 | 8 |
| Li F,2020^[37]^ | 1 | 1 | 0 | 1 | 1 | 1 | 1 | 1 | 7 |
| Shuanggang C,2020^[38]^ | 1 | 1 | 0 | 1 | 2 | 1 | 1 | 1 | 8 |
| Sun T,2020^[39]^ | 1 | 1 | 0 | 0 | 1 | 1 | 1 | 1 | 6 |
| Wu H,2020^[40]^ | 1 | 1 | 0 | 1 | 1 | 1 | 1 | 1 | 7 |
| Wu P,2020^[41]^ | 1 | 1 | 0 | 1 | 1 | 1 | 1 | 1 | 7 |
| Xu B,2020^[42]^ | 1 | 1 | 0 | 1 | 1 | 1 | 1 | 1 | 7 |
| Xu H,2020^[43]^ | 1 | 1 | 0 | 0 | 1 | 1 | 1 | 1 | 6 |
| Yang Y,2020^[44]^ | 1 | 1 | 0 | 1 | 1 | 1 | 1 | 1 | 7 |
| Cao F,2019^[45]^ | 1 | 1 | 0 | 1 | 2 | 1 | 1 | 1 | 8 |
| Cui H,2019^[46]^ | 1 | 1 | 0 | 0 | 1 | 1 | 1 | 1 | 6 |
| Fan W,2019^[47]^ | 1 | 1 | 0 | 1 | 1 | 1 | 1 | 1 | 7 |
| Li W,2019^[48]^ | 1 | 1 | 0 | 1 | 2 | 1 | 1 | 1 | 8 |
| Qiao X,2019^[50]^ | 1 | 1 | 0 | 0 | 1 | 1 | 1 | 1 | 6 |
| Qiu Z,2019^[51]^ |  |  |  |  |  |  |  |  |  |
| Shen R,2019^[52]^ | 1 | 1 | 0 | 1 | 1 | 1 | 1 | 1 | 7 |
| Wu F,2019^[54]^ | 1 | 1 | 0 | 0 | 1 | 1 | 1 | 1 | 6 |
| Xiao Q,2019^[56]^ | 1 | 1 | 0 | 1 | 1 | 1 | 1 | 1 | 7 |
| Xiong J,2019^[57]^ | 1 | 1 | 0 | 0 | 1 | 1 | 1 | 1 | 6 |
| Yang Z,2019^[59]^ | 1 | 1 | 0 | 1 | 2 | 1 | 1 | 1 | 8 |
| Zhu Y,2019^[61]^ | 1 | 1 | 0 | 0 | 1 | 1 | 1 | 1 | 6 |
| Chen S,2019^[62]^ | 1 | 1 | 0 | 1 | 2 | 1 | 1 | 1 | 8 |
| Huang R,2018^[64]^ | 1 | 1 | 0 | 0 | 1 | 1 | 1 | 1 | 6 |
| Wu J,2018^[65]^ | 1 | 1 | 0 | 1 | 1 | 1 | 1 | 1 | 7 |
| Lu W,2018^[69]^ | 1 | 1 | 0 | 1 | 2 | 1 | 1 | 1 | 8 |
