# Supplementary figures and images for: What Is the Most Suitable Agent Combined With Apatinib for Transarterial Chemoembolization Treatment in Advanced Hepatocellular Carcinoma Patients? A Systematic Review and Network Meta-analysis
Source: Front Oncol. 2022 May 25;12:887332. doi: 10.3389/fonc.2022.887332 (PMC9174538; doi:10.3389/fonc.2022.887332)

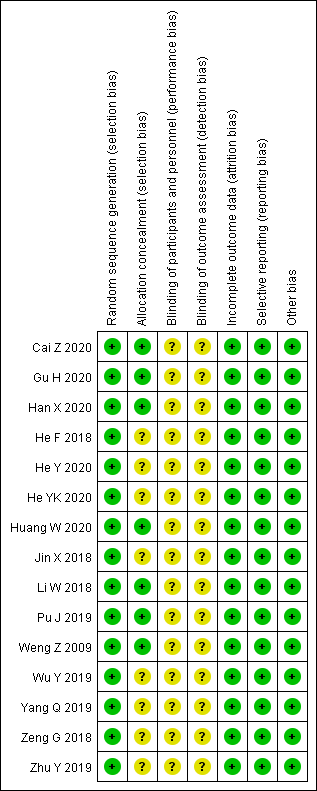

Supplement: Supplementary Figure 1 — Risk of bias summary Cochrane Collaboration’s tool. [file Image_1.png]

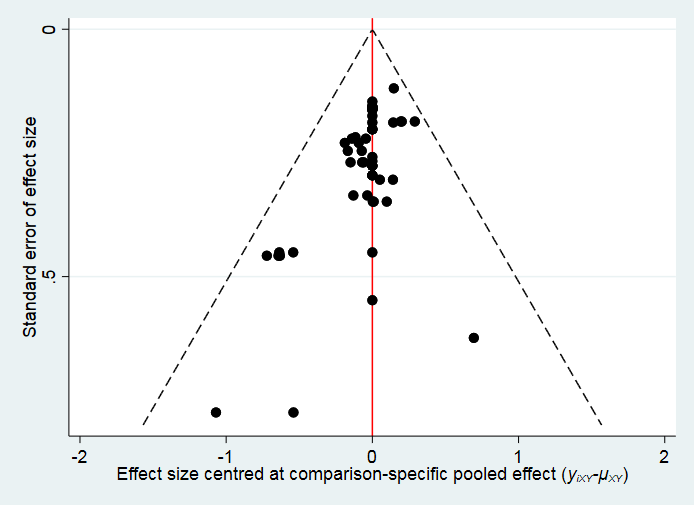

Supplement: Supplementary Figure 2 — Inconsistency plot of included studies with treatment response. [file Image_2.tif]
